# Cocaine-Induced Bronchial Laceration: A Rare Incidence

**DOI:** 10.7759/cureus.14252

**Published:** 2021-04-01

**Authors:** Rahul Dadhwal, Chinthaka P Bulathsinghala, Irfan Choudhry, Pahnwat T Taweesedt, Salim Surani

**Affiliations:** 1 Pulmonary Medicine, Corpus Christi Medical Center, Corpus Christi, USA; 2 Internal Medicine, University of Incarnate Word School of Osteopathic Medicine, San Antonio, USA; 3 Internal Medicine, Corpus Christi Medical Center, Corpus Christi, USA; 4 Internal Medicine, University of North Texas, Dallas, USA

**Keywords:** tracheobronchial injuries, bronchial laceration, cocaine, pneumomediastinum, bronchoscopy, intubation, multidisciplinary team

## Abstract

Tracheobronchial injuries are either traumatic or iatrogenic but can be lethal in a high dependency setting if not managed promptly. There are few reported cases of cocaine-induced airway damage and barotrauma due to thermal or ischemic injury and increased intra-alveolar pressure. We present a sui generis case of cocaine-induced bronchial laceration with pneumomediastinum which was challenging to diagnose based on the patient’s recent history of hospitalization, as well as the patient’s reluctance to share the history of cocaine use. The patient was successfully managed conservatively. Here, we discuss the mechanism involved and the various treatment options available, along with the role of early involvement of the multidisciplinary team to deliver the best possible outcome.

## Introduction

Tracheobronchial injury is an infrequent injury usually involving the trachea, right or left main stem bronchi, and is associated with significant morbidity and mortality [1]. Tracheobronchial injuries are mostly caused by either trauma or are iatrogenic [2]. The use of cocaine causes numerous airway complications ranging from thermal injury, nasal septum perforation, primary cervical emphysema, to spontaneous pneumomediastinum, but cocaine-induced bronchial laceration leading to pneumomediastinum is a rare phenomenon.

While primary spontaneous pneumomediastinum is usually benign and self-limiting in nature, secondary pneumomediastinum more often has serious outcomes that require surgical intervention based on their causal factors [3]. Universally acknowledged pathophysiological explanation for primary pneumomediastinum is alveolar rupture due to barotrauma causing air leakage which leads to free air in the mediastinal plane [4], whereas secondary causes include trauma, infection, or being iatrogenic [3]. Here, we present a unique case of cocaine-induced bronchial laceration and discuss the mechanism behind it. The patient was managed conservatively after a detailed discussion with the multidisciplinary team, and the approach led to complete resolution of the laceration.

## Case presentation

**Figure 1:**
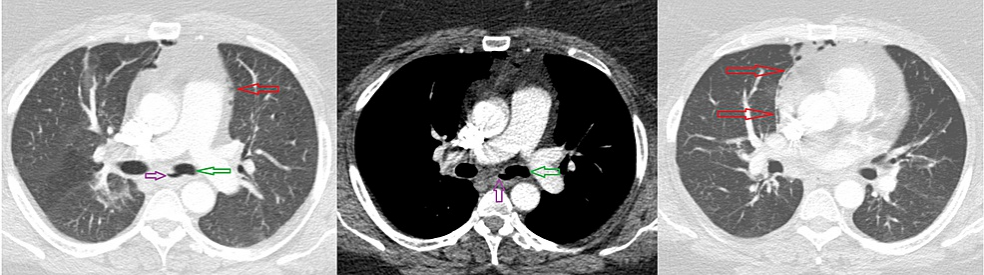
Computed tomography showing pneumomediastinum (red arrows) which may be secondary to a connection between the esophagus (purple arrows) and left main stem bronchus (green arrows).

A 44-year-old Hispanic female with a history of human immunodeficiency virus infection presented to the emergency department (ED) with dyspnea on exertion for four hours. The patient had undergone an elective hysterectomy a week before presentation, where the patient, as per anesthesia records, underwent uneventful intubation without the use of a bougie, with the endotracheal tube at the 22-cm mark at the lip level and was successfully extubated after the procedure. The patient’s hospital course was benign, and she denied any complaints, following which she was discharged home two days later. In the ED, the patient underwent further questioning and admitted to snorting “crack cocaine” several hours before the presentation. A computed tomography angiogram of the chest did not reveal a pulmonary embolism; however, a questionable pneumomediastinum between the esophagus and left main bronchus could not be ruled out (Figure 1).

**Figure 2:**
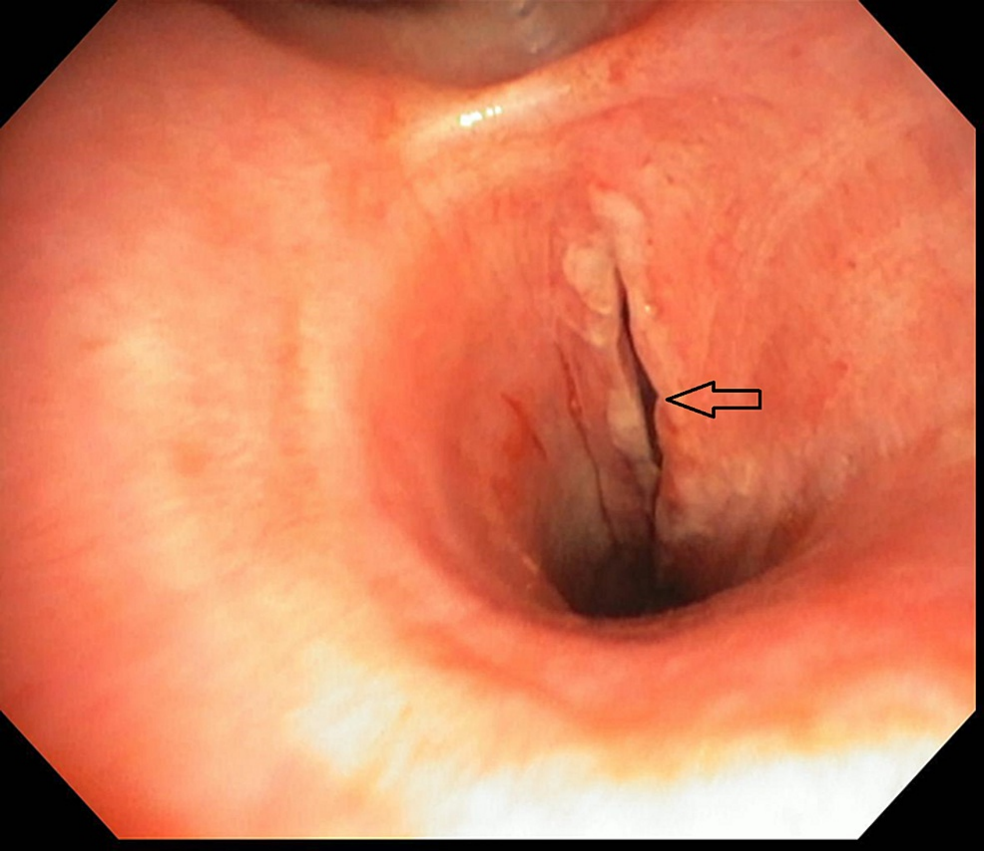
Bronchoscopy image showing left main stem bronchial mucosal tear (arrow).

Therefore, several hours after the admission, a bronchoscopy was performed which revealed a bronchial mucosal laceration measuring 1.5 inches at the 24.5-cm mark from the lip level (Figure 2). The rest of the bronchoscopy was unremarkable.

**Figure 3:**
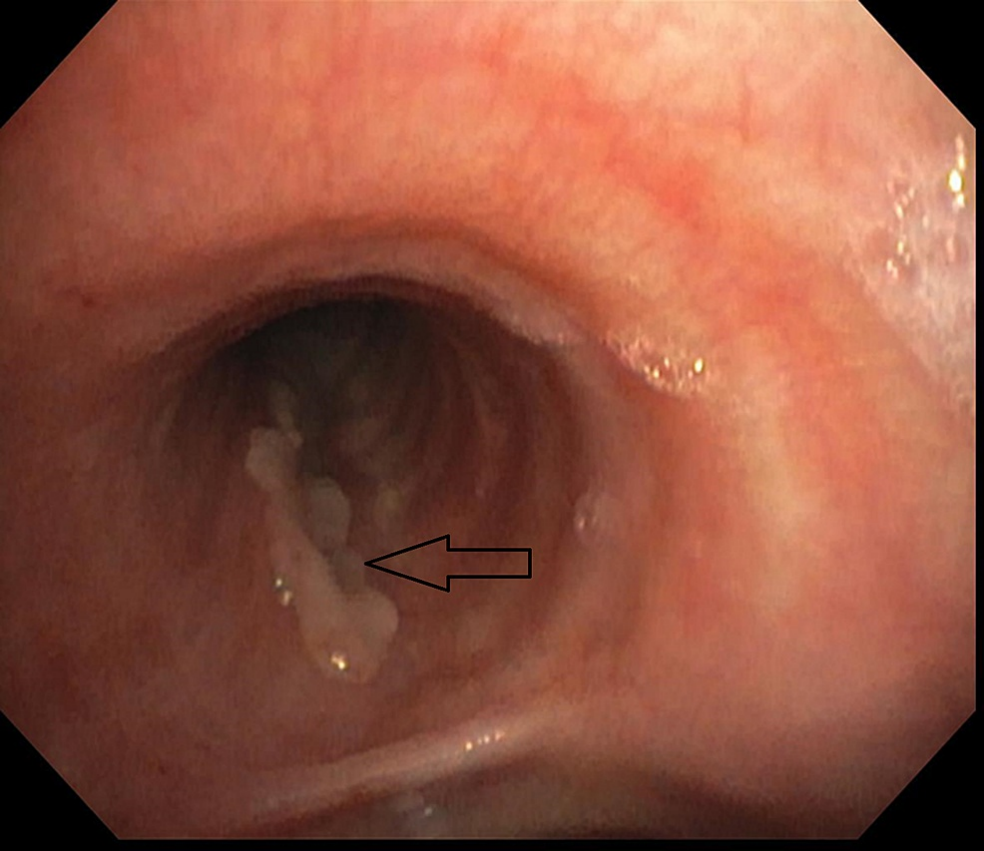
Bronchoscopy image showing healed left main stem bronchial mucosal tear (arrow).

Cardiothoracic surgery was consulted and conservative management was recommended. The patient was monitored in the hospital for five days without any compromise in her breathing or swallowing capabilities. A repeat bronchoscopy was performed on day five of the admission, which showed a healed left main stem bronchial mucosal tear (Figure 3).

## Discussion

Cocaine, or benzoylmethylecgonine (C_17_H_21_NO_4_), is an illegal powerful addictive stimulant drug that is a naturally occurring alkaloid extracted from the leaves of the *Erythroxylon coca* plant after a complex chemical process and is available in four forms, namely, hydrochloride salt, freebase, crack, and bazuco [5,6].

The hydrochloride form is a fine white powder that is made by dissolving the alkaloid in hydrochloric acid which decomposes. It is generally administered intranasally by “snorting,” orally by rubbing it on the gums, or intravenously by turning it into a liquid, sometimes combining it with heroin, called a speedball. This form is not heat stable and cannot be smoked. Dissolving cocaine hydrochloride in water and then adding a base and a solvent gives us the freebase form, whereas dissolving cocaine hydrochloride in water with sodium bicarbonate makes the resultant substrate heat stable, known as crack, which can be smoked and is named after the sound it makes when heated [7-9]. The crude extract of coca leaves when mixed with other substances results in a highly adulterated form of cocaine paste called bazuco or “bazooka.” Street samples of cocaine consist of many adulterants such local anesthetics (benzocaine, lidocaine), toxins (strychnine, quinine), sugars (sucrose, mannitol, lactose), inert compounds (inositol, cornstarch, talc), stimulants (ephedrine, caffeine), as well as other substances (e.g., calcium, plaster, flour, aspirin, gasoline, sulfuric acid, sand), resulting in additional toxicity [10].

Several cases have been reported of cocaine-induced pneumomediastinum via nasal insufflation, “snorting,” or smoking inhalation [8,11,12]. The pathophysiology described is usually related to intra-alveolar barotrauma caused by either forced inhalation with a closed mouth and nostril followed by the Valsalva maneuver to ensure maximum absorption across the alveolar-capillary membrane or by severe cough triggered by cocaine smoke inhalation, leading to increased intrathoracic pressure, as well as causing alveolar rupture leading to pneumothorax, pneumomediastinum, pneumopericardium, or subcutaneous emphysema [4,8,13]. However, the pathophysiology of barotrauma does not provide a plausible causal explanation to the above-mentioned case of left main stem bronchial mucosal laceration. The topical effect of cocaine directly on mucosal linings could be a more persuasive explanation. Repeated nasal insufflation of cocaine not only causes mucosal lesion of the nose and upper airways extending to the perichondrium but can also cause ischemic necrosis and perforation due to cocaine’s property of being a potent vasoconstrictor [14,15]. Thermal airway injury can also occur secondarily from either inhalation injury caused by the chemical byproducts or due to combustion of highly inflammable solvents (impurities) used during the production process [8,16].

**Figure 4:**
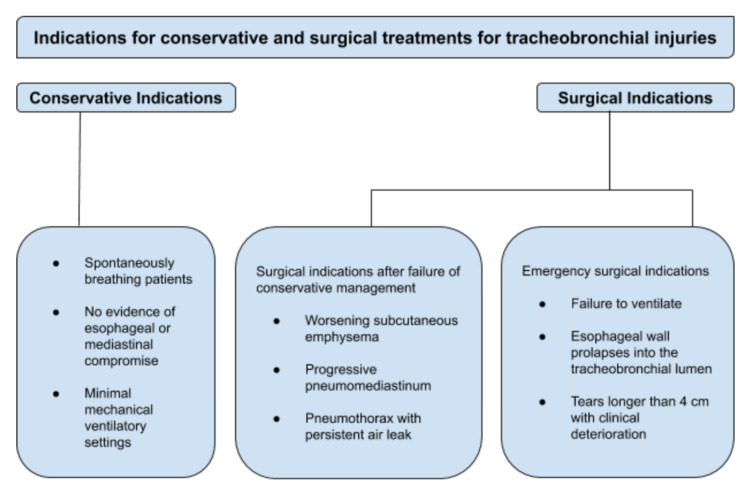
Approach for conservative versus surgical treatment modalities for tracheobronchial injuries.

An accurate diagnosis of tracheobronchial tear is often made with a high degree of clinical suspicion with the aid of radiological findings or bronchoscopy findings in suspected cases. However, the missed diagnosis of tracheobronchial injuries can lead to significant morbidity and mortality. The crucial aspect of emergency management of a tracheobronchial laceration is to safely secure the airway, for which fiberoptic bronchoscopic intubation is the ideal choice as it can help suction clots and secretions, does not require neck extension, and ensures that the placement of the cuff is distal to the injured airway, and if the injury is at the level of carina or one of the main bronchi, then positioning the tube into the uninjured bronchus could be achieved under endoscopic guidance, thereby helping in the initiation of single lung ventilation. However, if the patient is bleeding actively, has distal airway collapse, or is restless, unstable, and uncooperative with hemodynamic compromise, it makes the procedure exceedingly challenging. As it can remove clots and secretions more effectively, rigid bronchoscopy under inhalational anesthesia is considered ideal in such conditions; however, as it requires neck extension, it is contraindicated even in suspected cases of cervical spine injury [1]. Surgical repair may be needed in severe cases. During ventilation in such patients, high airway or positive end-expiratory pressures must be avoided and extracorporeal membrane oxygenation must be considered for patients who are unable to ventilate as a bridge to recovery or a conclusive repair either bronchoscopically or surgically [17,18]. The best way to manage a patient with a tracheobronchial tear is to set up a robust multidisciplinary professional team to deliver the best outcome for the patient [19]. The approach for conservative versus surgical treatment modalities for tracheobronchial injuries is presented in Figure 4 in detail [20].

In our patient, the conservative treatment modality was preferable due to her stable clinical status, laceration size measuring less than 4 cm, ability to take spontaneous breaths, and absence of definitive clinical signs of mediastinitis.

## Conclusions

A cocaine-induced bronchial mucosal laceration is a rare presentation which if left undiagnosed and without prompt and aggressive conservative management can become a life-threatening situation leading to significant morbidity and mortality. An exhaustive assessment of patients on the basis of their clinical status, types of comorbidities, size and site of perforation, mode of presentation, duration from perforation to presentation, and early involvement of a multidisciplinary team is the right way to approach to achieve the best possible outcome for the patients.
